# Fire Frequency Driven Increases in Burn Heterogeneity Promote Microbial Beta Diversity: A Test of the Pyrodiversity‐Biodiversity Hypothesis

**DOI:** 10.1111/mec.17756

**Published:** 2025-04-05

**Authors:** Jacob R. Hopkins, Alison E. Bennett, Thomas P. McKenna

**Affiliations:** ^1^ Evolution, Ecology, & Organismal Biology The Ohio State University Columbus Ohio USA; ^2^ Kansas Biological Survey and Center for Ecological Research University of Kansas Lawrence Kansas USA

**Keywords:** burn pattern, diversity, fire, fire‐microbe, microbes, pyrodiversity

## Abstract

Fire is a common ecological disturbance that structures terrestrial ecosystems and biological communities. The ability of fires to contribute to ecosystem heterogeneity has been termed pyrodiversity and has been directly linked to biodiversity (i.e., the pyrodiversity–biodiversity hypothesis). Since climate change models predict increases in fire frequency, understanding how fire pyrodiversity influences soil microbes is important for predicting how ecosystems will respond to fire regime changes. Here we tested how fire frequency‐driven changes in burn patterns (i.e., pyrodiversity) influenced soil microbial communities and diversity. We assessed pyrodiversity effects on soil microbes by manipulating fire frequency (annual vs. biennial fires) in a tallgrass prairie restoration and evaluating how changes in burn patterns influenced microbial communities (bacteria and fungi). Annual burns produced more heterogeneous burn patterns (higher pyrodiversity) that were linked to shifts in fungal and bacterial community composition. While fire frequency did not influence microbial (bacteria and fungi) alpha diversity, beta diversity did increase with pyrodiversity. Changes in fungal community composition were not linked to burn patterns, suggesting that pyrodiversity effects on other ecosystem components (e.g., plants and soil characteristics) influenced fungal community dynamics and the greater beta diversity observed in the annually burned plots. Shifts in bacterial community composition were linked to variation in higher severity burn pattern components (grey and white ash), suggesting that thermotolerance contributed to the observed changes in bacterial community composition and lower beta diversity in the biennially burned plots. This demonstrates that fire frequency‐driven increases in pyrodiversity augment biodiversity and may influence productivity in fire‐prone ecosystems.

## 1 Introduction

Fire is a common ecological disturbance that structures many terrestrial ecosystems and drives long‐term evolutionary patterns in biological communities (Archibald et al. 2013; McLauchlan et al. 2020). Fire effects on above and belowground ecosystem components can be quite heterogeneous (Whelan 1995; Neary and Leonard 2020; Zhang et al. 2021). Overtime, these local variations in fire frequency and severity (damage to plant and soil biota, and organic matter loss) contribute to “burn mosaics” of different abiotic conditions and greater habitat diversity (i.e., pyrodiversity; Agee 1998; Fox et al. 2022; Steel et al. 2021). Since greater habitat diversity benefits greater numbers of organisms, it follows that heterogeneity in burn patterns (the pattern of fire occurrence and severity effects on soils) will boost biodiversity (pyrodiversity–biodiversity hypothesis; He et al. 2019; Jones and Tingley 2022). Because climate change models predict changes in fire regimes (e.g., increased fire frequency and longer fire seasons; Halofsky et al. 2020; Jolly et al. 2015; Jones, Abatzoglou et al. 2022), it is important that we understand how variation in fire regime components like fire frequency influences pyrodiversity–biodiversity relationships (Jones, Ayars et al. 2022).

Pyrodiversity–biodiversity relationships are driven by interactions between fire, plant fuels, and soils (Archibald et al. 2018; Pausas and Bond 2019). When fires are too infrequent, plant fuels build up and produce more continuous fuel loads that favour higher severity and more uniform fires (Kolden 2019; Roos et al. 2020). When fire frequency increases however, overall fuel loads are reduced and local variation in available fuels becomes patchier (Kalies and Kent 2016; Oliveira et al. 2021). These relationships between fire frequency and severity are exemplified in the historical fire return intervals of pyrophilic ecosystems like tallgrass prairies (5–10 years), longleaf pine savannas (5 years), cerrados (< 10 years) and Ponderosa pine forests (13 years) where fire suppression has contributed to severe fires outside the historic norm (relative to the historical pattern of frequent, lower severity fires) and/or shifts in ecosystem state (Mistry et al. 2005; Pyke et al. 2010; Kalies and Kent 2016; Roos et al. 2020; Abrahamson 2023). This local variation in fuel conditions produces spatial differences in fire severity that over time contribute to greater ecosystem heterogeneity or pyrodiversity (Jensen et al. 2001; Harris et al. 2019; Povak et al. 2023). For example, differences in fire severity can contribute to spatial variation in nutrient availability (N decreases with severity; and P increases with severity; Certini 2005; Butler et al. 2018; Alcañiz et al. 2018), soil hydrophobicity (Huffman et al. 2001; Letey 2001) and soil oxidative stress (Fu et al. 2018; Yan et al. 2022). This spatial variation in soil conditions then influences post‐fire plant communities since pyrophilic plant taxa often demonstrate specificity for different fire frequencies and post‐fire conditions (Parra and Moreno 2017; Revillini et al. 2022; Hopkins, Huffman et al. 2023; Hopkins and Bennett 2024b). While the pyrodiversity–biodiversity hypothesis is supported by recent work with plant communities (Ponisio et al. 2016; Miller and Safford 2020), the close associations between plants and soil microbes suggest that pyrodiversity may also influence belowground communities and biodiversity (Jones and Tingley 2022; Fox et al. 2022; David et al. 2023).

Soil microbial responses to fire are not ubiquitous and demonstrate a high degree of variability in adaptations to surviving fire and fire effects on soil conditions (Certini et al. 2021; Fox et al. 2022). Pyrophilic soil microbes (bacteria and fungi) possess distinct trait profiles and employ different strategies that allow them to utilise different post‐fire niches (Johnson et al. 2023; Hopkins and Bennett 2024a). For example, microbial taxa that survive the passage of fire (i.e., fire resistant taxa) often possess heat resistant cell walls (Peay et al. 2009; Balogh et al. 2013) and produce protective, heat‐resistant pigmentation (Pérez‐Izquierdo et al. 2020; Rajamani et al. 2021; Hewitt et al. 2022) that helps them survive the direct effects of fire. Alternatively, other pyrophilic microbes demonstrate post‐fire strategies that either allow them to rapidly disperse into burned patches (Kobziar et al. 2018; Barbour et al. 2023), utilise post‐fire nutrient flushes (Pugh 1980; Hopkins, Semenova‐Nelsen et al. 2023) or tolerate stressful post‐fire conditions (e.g., xeric soil, UV exposure, oxidative stress; Wynn‐Williams et al. 2002; Cordero and Casadevall 2017; Hopkins and Bennett 2023). Therefore, systems with greater levels of pyrodiversity should support greater soil microbial diversity. Despite the potential for pyrodiversity to modify microbial communities and biodiversity, very few studies have considered soil microbial responses to pyrodiversity (Jones and Tingley 2022).

Soil microbes influence fuel loads through their roles as decomposers, plant pathogens and mutualists (Smith and Read 2010; Pausas and Ribeiro 2013; Hopkins et al. 2020; Gibb et al. 2022); therefore, understanding microbial pyrodiversity–biodiversity relationships is critical to our knowledge of the above and belowground processes that structure fire regimes. Furthermore, soil microbes provide unique opportunities for identifying the relative importance of pyrodiversity components (e.g., direct vs. indirect effects of fire) on biodiversity due to their short generation times and high dispersal rates that allow for differentiation between stochastic and deterministic processes that influence community assembly (Barberán et al. 2014). Many bacterial generation times last from a few minutes to a few hours (Gibson et al. 2018; Weissman et al. 2021), which can allow for rapid assessment of how local variation in fire severity influences soil biodiversity. Whereas fungi, which have relatively longer generation times (hours to months; Rousk and Bååth 2011; Gostinčar et al. 2022) and stronger associations with plant hosts (Gao et al. 2013; Vos et al. 2013; Mommer et al. 2018), allow for assessment of how longer‐term effects of pyrodiversity influence soil biota. Consequently, soil fungi and bacteria are valuable bioindicators (i.e., organisms used to assess ecosystem health) for assessing temporal variation in pyrodiversity–biodiversity relationships.

We tested how fire frequency driven changes in burn patterns (i.e., pyrodiversity) influenced soil microbial communities and diversity. Burn patterns, soil fungal and soil bacterial communities were sampled after prescribed fire in a tallgrass prairie restoration that experimentally manipulates fire frequency (annual and biennial fires). This allowed us to test three questions: (1) does fire frequency determine the heterogeneity of burn patterns? (2) Do changes in fire frequency alter soil bacterial and fungal communities (compositional shifts and altered abundances of fire related taxa) and diversity patterns (alpha and beta diversity)? And (3) are fire frequency effects on burn heterogeneity (degree of within group variation in burn pattern components like ash colour, bare ground, and unburned fuels) linked to post‐fire changes in soil microbial communities? We hypothesised that increases in fire frequency would produce more stochastic burn patterns, changes in microbial community composition, and greater microbial diversity. We further hypothesised that burn pattern heterogeneity would be correlated with increases in soil microbial diversity.

## 2 Materials and Methods

### 2.1 Study System

This study took place in a tallgrass prairie restoration at the Perennial Agriculture Project Field Station near Lawrence, KS, USA (39°0′ 6.9156″ N, 95°19′ 10.4376″ W). The growing season at this site stretches from April to September, with an average annual rainfall of 900–1000 mm (majority between March and August). Prior to restoration, this site was dominated by *Bromus inermis* (invasive in North America) and had not been planted for at least one decade. In March 2021, 40, five m^2^ square plots were established with 5 m buffer zones between each plot. Plots were planted with a regional tallgrass prairie seed mix from Hamilton Native Outpost (Elk Creek, MO). The seed mix included grasses (7 species), forbs (22 species) and legumes (3 species) such as *Schizachyrium scoparium* , *Bouteloua curtipendula* , *Panicum capillare* , *Rudbeckia hirta* , *Liatris pycnostachya* , *Echinacea pallida* , *Solidago nemoralis* , *Coreopsis grandiflora* , *C. tinctoria* and *Chamaecrista fasciculata* (See Table S1 for full species list and site pictures). Of the 34 seeded species, 27 have been found in the site (84%). Following planting, fire regime treatments were established in October 2021 (Figure S1). Fire regime treatments are based on three‐year intervals that manipulate fire season (e.g., spring vs. fall) and fire frequency (annual, biennial, triennial, no fire). In this study, only the spring burned, annual (*n* = 5) and biennial (*n* = 5) plots were utilised. The annually burned plots are expected to have lower severity and more heterogeneous fires due to repeated fuel reductions from annual burns. Prior to prescribed burns, firebreaks are mowed between plots. Each plot is burned with a drip torch by igniting backing fires along the leeward side, followed by fires along the windward side. Fires burn on average for 5–10 min, with fuel combustion estimated around 75%–80%. Prescribed fire in this system is less severe than grassland wildfires due to reliable fire‐driven reductions in plant fuels that limit fire severity (Stubbendieck et al. 2007). Some invasive species like *B. inermis* are occasionally found in the plots; however, the seeded species dominate the entire site. When found, woody taxa like *Gleditsia triacanthos* are removed from the plots.

### 2.2 Burn Pattern Quantification

**FIGURE 1:**
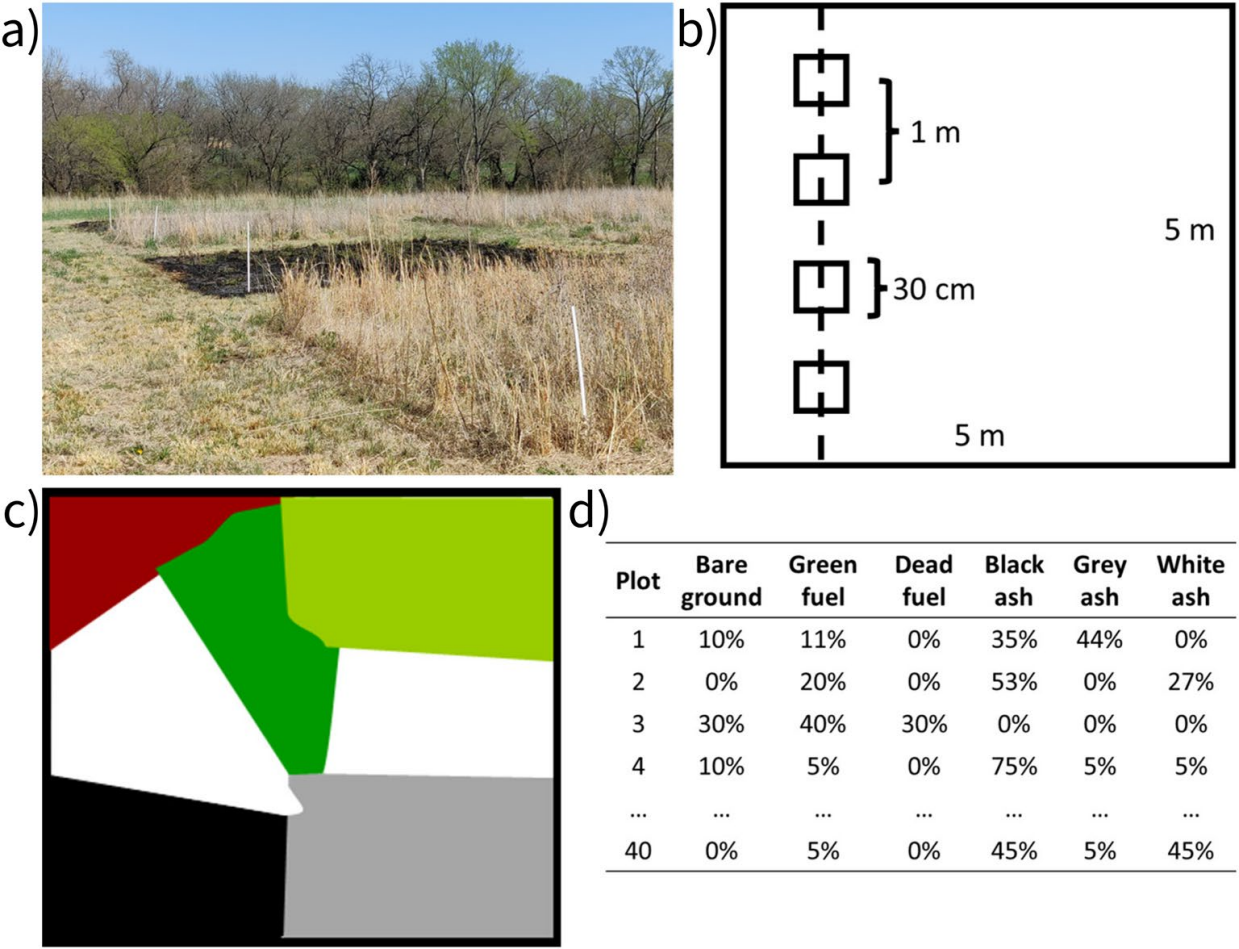
Field assessment of burn patterns. (a) Annual and biennial fire treatment plots were burned in April 2024. (b) Following fires, transects were randomly established in each plot (*n* = 10). Along each transect four, 900 cm^2^ square sub‐plots were placed 1 m apart. (c) Percent cover for the following burn pattern components were estimated: Bare ground (brown), green‐unburned fuels (dark green), dead‐unburned fuels (light green), black ash (black; low severity burn), grey ash (grey; moderate severity burn) and white ash (white; high severity burn). (d) Percent cover data for each plot (summing to 100%) were recorded and analysed as compositional data. Note that the six burn pattern components together represent the overall burn pattern for the plot.

Immediately after April 2024 prescribed burns (Figure 1a), a 5 m transect was randomly placed in each plot (Figure 1b). Every 1 m (*n* = 4) along the transect, a 900 cm^2^ square grid was placed on the ash layer, and burn patterns within the grid were estimated using percent cover methods (Figure 1c). Briefly, percent cover (summing to 100%) of unburned green fuels (live), unburned dead fuels, black ash (low‐moderate fire severity), grey ash (moderate fire severity), white ash (high severity) and bare ground were quantified (Pereira et al. 2012). This produced a total of 40 burn pattern measurements (20 annual and 20 biennial; Figure 1d).

### 2.3 Microbial Field Sampling

Immediately after fire, at each transect point, two soil cores (0–10 cm depth; 50 mL total) were collected using a soil corer (2 cm diameter) and homogenised within a sterile sample bag. The soil corer was sterilised with soapy water and ethanol between samples. Microbial soil samples were kept on ice in the field and then stored at −20°C until DNA extractions. This produced a total of 40 soil microbial samples for downstream DNA analysis.

### 2.4 DNA Extraction, Library Preparation and Sequencing

DNA was extracted from 0.25 g of each soil microbial sample using a Qiagen DNeasy PowerSoil Pro Kit (Hilden, Germany) following the manufacturer's instructions. Extracted DNA was then sent to the North Carolina State Genomic Sciences Laboratory for library preparation and sequencing. Fungal DNA libraries were prepared using the ITS1‐F and ITS2 primer pair (Kõljalg et al. 2013) for the initial PCR reaction, followed by PCR clean‐up with Agencourt Ampure XP Beads (Beckman Coulter, Brea, CA, USA) and 80% EtOH, followed by a second PCR where Illumina barcodes were added (Nextera Indices, Illumina, San Diego, CA, USA), followed by another PCR clean‐up step, pooling at equimolar concentrations, and a quality control check with a Bioanalyzer DNA 1000 chip (Agilent, Santa Clara, CA, USA). Bacterial DNA libraries were prepared similarly; however, the S‐D‐Bact‐0341‐b‐S‐17 (forward) and S‐D‐Bact‐0785‐b‐A18 (reverse) primers were used (Klindworth et al. 2013). ITS and 16S libraries were sequenced using the Illumina MiSeq platform with V3 chemistry (San Diego, CA, USA). Sequence data were deposited in the GenBank Sequence Read Archive (SRA; PRJNA1240331—bacteria; PRJNA1241203—fungi).

### 2.5 Bioinformatics

Raw sequence data were analysed using Qiime 2 version 2022.2 following methods described in (Bolyen et al. 2019). Quality and barcode filtering resulted in approximately 8.5 M reads for ITS data (40 samples) and 13.6 M reads for 16S data (40 samples). Two samples were removed from the 16S data due to low relative read counts (less than half the average of all other samples). Illumina barcodes were trimmed off paired reads using cutadapt (Martin 2011), then combined with dada2 (Callahan et al. 2016). The UNITE fungal ITS reference database v9 “dynamic” (Abarenkov et al. 2010) and SILVA 138 99% OTU database (Quast et al. 2013) were used to train naïve bayes classifiers, which then categorised reads in amplicon sequence variants (ASVs) and assigned taxonomic identities. ASVs with fewer than five reads were removed. This resulted in 16,813 bacterial ASVs and 8006 fungal ASVs. Bioinformatics scripts are included in the supplement.

### 2.6 Statistical Analyses

All analyses were performed in R v. 4.3.2 (R Core Team 2022). Dissimilarity matrices were produced for burn pattern (Aitchison), fungi (Bray‐Curtis) and bacteria (Bray‐Curtis) with the dist function in the Vegan package (Oksanen et al. 2007). Principal coordinates analysis (PCoA) was then used to create ordinations for the burn pattern and microbial community data using the prcomp function. For burn pattern ordinations, the first three axes were retained for downstream analysis (variance explained sums to 75.6%), whereas for microbial ordinations broken stick models were used to determine the number of axes to retain for fungi and bacteria (first three principle coordinates axes for both fungi and bacteria).

To test for differences in burn patterns (overall percent cover of burn pattern components; Figure 1d), burn data were converted into a closed Aitchison compositional object (summing to 100%) using the acomp function in the compositions package (van den Boogaart and Tolosana‐Delgado 2008). The burn pattern compositional object was then transformed using an isometric log ratios (linearises compositional data) and included in an analysis of variance (ANOVA) with field column (controls for locational effects) and fire regime treatment as fixed effects. Compositional means for burn pattern components (bare ground, green unburned fuels, dead unburned fuels, black ash, grey ash and white ash) were then extracted by back transforming model coefficients with the ilrInv function for accurate graphical representation. To assess differences in pyrodiversity (heterogeneity of burn pattern components) between fire regime treatments, beta dispersion of the burn patterns was assessed using the betadisper and anova functions. Beta dispersion allows for comparison of average distance to group centroids in ordination space, and in this usage, greater average distance means greater heterogeneity or pyrodiversity (Anderson 2006).

To test for differences in microbial community composition between fire regime treatments (annual vs. biennial fires), PERMANOVAs (adonis2 function; 10,000 permutations) that accounted for location effects were used. Models included fire regime treatment as a fixed effect, while the strata argument was used to handle the plot column as a random effect. To assess differences in beta dispersion (stochasticity and beta diversity metric) between fire regime treatments, beta dispersion was assessed using the betadisper function. When applied to biological community data, beta dispersion measures the degree of similarity (or stochasticity) in community composition between treatment groups (Anderson et al. 2006). If beta dispersion is higher for a group (i.e., more stochastic), then this means that beta diversity is higher for that group. In the context of this study, we predicted that beta dispersion values would be lower in biennially burned plots since fires were expected to be more homogenous in nature and therefore have a more deterministic rather than stochastic effect on microbial communities. To assess deterministic effects on microbial community composition, correlations between ash patterns, microbial diversity (see below) and microbial community composition were assessed using the envfit function (10,000 permutations). To identify taxa indicative of annual and biennially burned fire regime treatments, the ALDEx2 package (Fernandes et al. 2013) was used to detect differential ASV expression. For ALDEx2 analyses, only samples with Wilcoxon test *p*‐values less than 0.001 were retained to correct for multiple comparisons.

Microbial diversity for fungi and bacteria was calculated using the diversity function (Inverse Simpson Metric), which accounts for both abundance and evenness. To test for differences in alpha diversity between fire regime treatments, type III linear mixed effect (LMER) models were used (lmer function, lme4 package; Bates et al. 2015). After model creation, type III sums of squares were extracted and fire regime effects were tested using the emmeans package (Lenth 2018). Diversity LMERs included plot column as a random effect and fire regime treatment as a fixed effect.

## 3 Results

**FIGURE 2:**
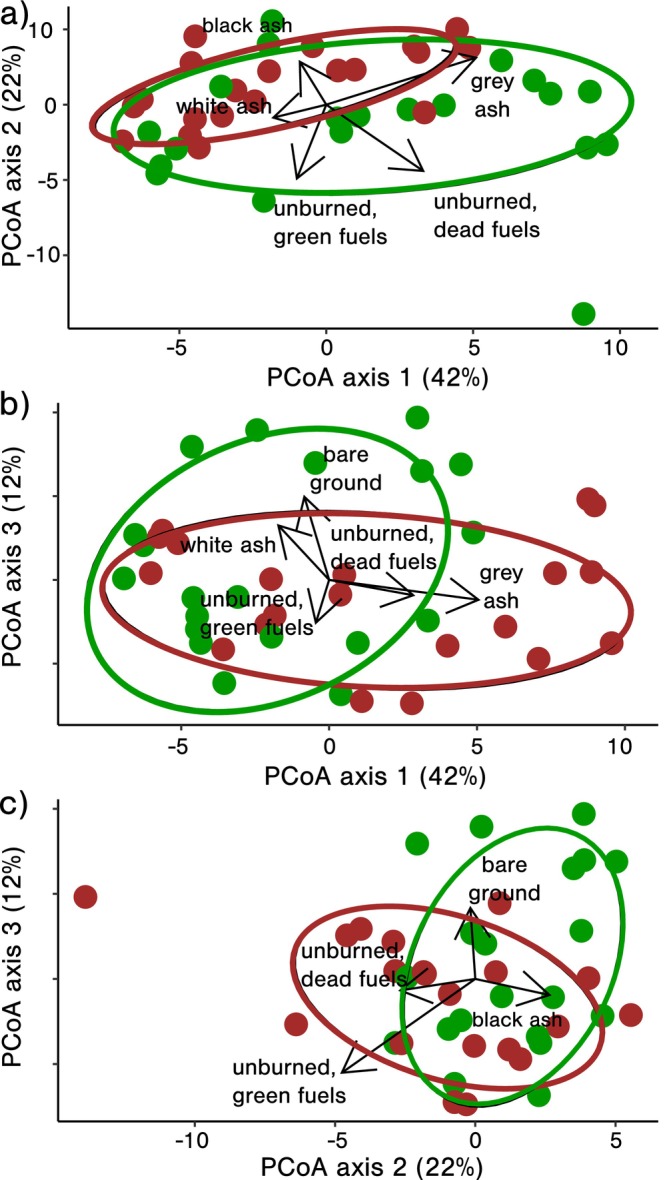
Fire regime effects on burn patterns. Ellipses correspond with 75% confidence intervals for annual (green) and biennial (brown) fire frequency regime treatments. Superimposed arrows correspond with burn pattern components correlated with overall burn patterns (*p* < 0.05). Panels (a) (axes 1 & 2), (b) (axes 1 & 3) and (c) (axes 2 & 3) show the first three PCoA axes and explain 76% of total variation in burn pattern data.

**FIGURE 3:**
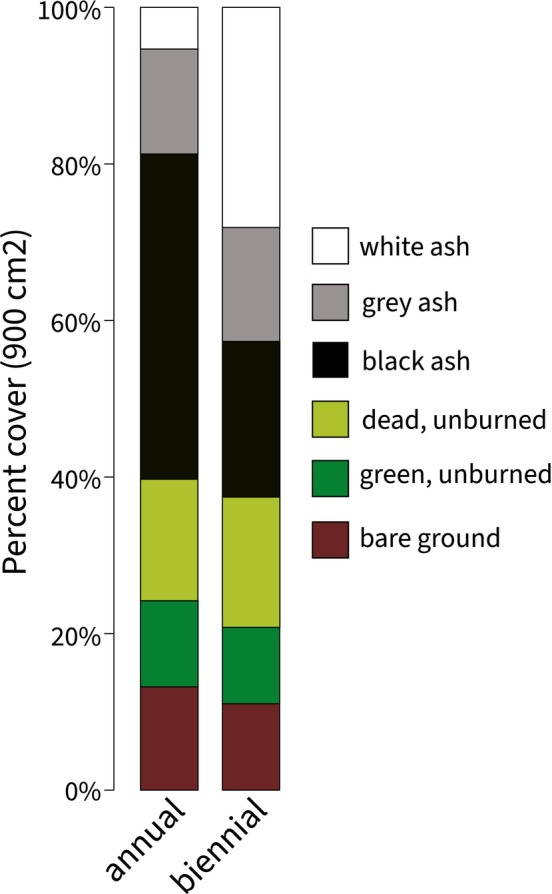
Burn pattern compositions for annual and biennial fire regime treatments. Bar segments correspond with compositional means of burn pattern components.

**FIGURE 4:**
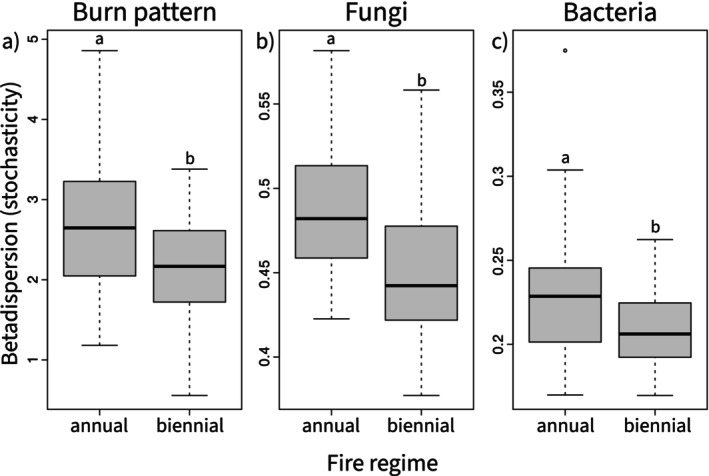
Fire regime effects on burn pattern and microbial beta dispersion (stochasticity/beta diversity). Annual versus biennial fire produced (a) burn patterns that were more heterogeneous and higher levels of (b) fungal and (c) bacterial beta diversity.

*Fire regime effects on burn patterns*: Fire regime treatments produced different burn patterns (*F*_1,34_ = 2.5, *p* = 0.05; Table 1 and Figures 2a–c, 3). Burn patterns in annually burned plots were more stochastic (*F*_1,38_ = 5.3, *p* = 0.03; Table 2 and Figure 4a) and had 21.6% more black ash (low severity) than biennially burned plots. Biennial burn patterns, however, were more uniform and had 22.8% more white ash (high severity) relative to annually burned plots. To summarise, differences in fire frequency produced variation in burn patterns, with higher fire frequency driving greater burn heterogeneity and lower severity fires.

**TABLE 1:** Compositional ANOVA table for fire frequency effects on burn patterns. Compositional means for each treatment are presented below ANOVA results.

	D.F.	Pillai	*F*	Numerator D.F.	Denominator D.F.	*p*
Intercept	1	0.64	10.65	5	30	< 0.001***
Field column	4	0.84	1.76	20	132	0.03*
Fire regime	1	0.3	2.53	5	30	0.05*
Residuals	34					

Means	Bare ground	Unburned, green fuels	Unburned, dead fuels	Black ash	Grey ash	White ash
Annual fire	13.2%	11%	15.5%	41.5%	13.4%	5.3%
Biennial fire	11%	9.8%	16.6%	19.9%	14.6%	28.1%

*Note: p* < 0.1, *, *p* < 0.05; **, *p* < 0.01; ***, *p* < 0.001.

**TABLE 2:** Beta dispersion tables for fire frequency effects on burn patterns and soil microbial communities.

Model term	D.F.	Sum Sqr.	Mean Sqr.	*F*	*p*	Sum Sqr.	Mean Sqr.	*F*	*p*	Sum Sqr.	Mean Sqr.	*F*	*p*
		Burn pattern				Fungi				Bacteria			
Fire regime	1	3.1	3.1	5.3	0.03*	0.01	0.01	6.2	0.02*	0.01	0.01	4.5	0.04*
Residuals	38	23	0.6			0.08	0.002			0.05	0.001		

*Note:* *p < 0.05.

**FIGURE 5:**
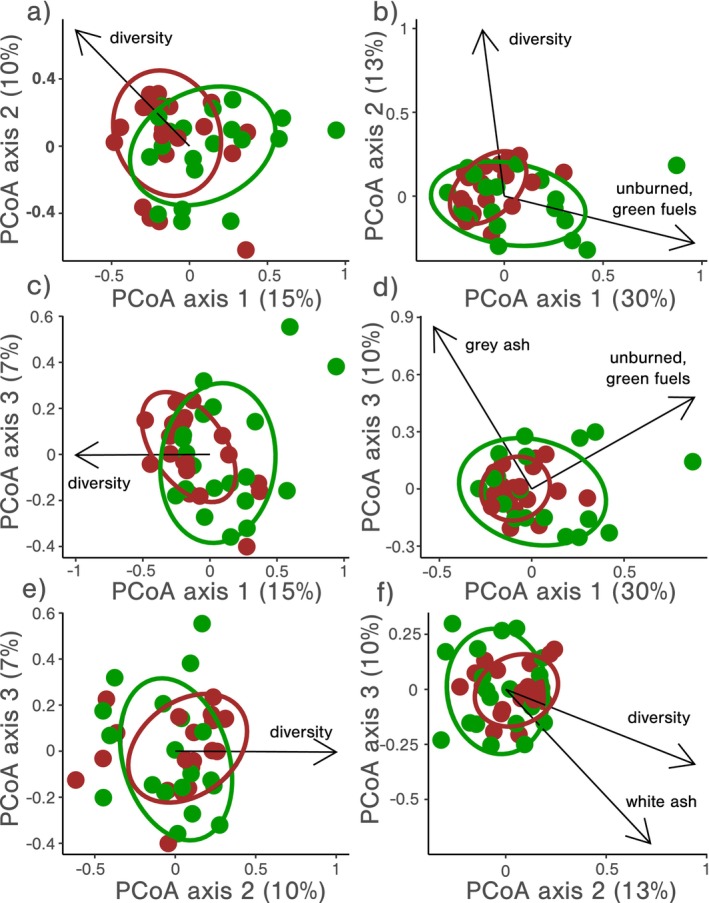
Fire frequency regime treatment effects on soil microbial communities. Ellipses correspond with 75% confidence intervals for annual (green) and biennial (brown) fire frequency regime treatments. Superimposed arrows correspond with burn pattern and diversity components correlated with microbial community composition (*p* < 0.05). Panels a, c, and e, represent fungal community PCoA axes (32% of total variation). Panels b, d and f, represent bacterial community PCoA axes (53% of total variation).

**FIGURE 6:**
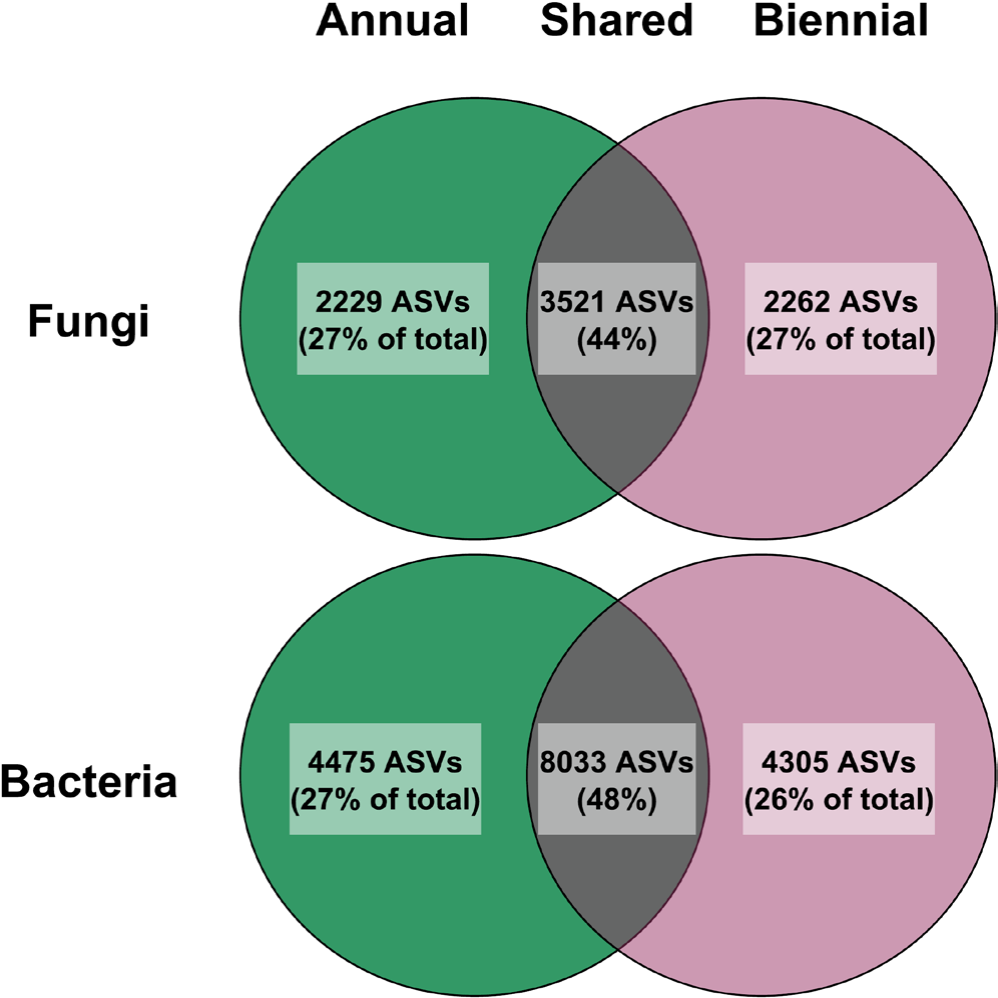
Number of unique and shared ASVs for annual and biennial fire frequency regime treatments.

*Fire regime effects on fungal communities*: Fire regime treatments produced differences in soil fungal communities (*F*_1,38_ = 1.42, *p* = 0.009; Table 3 and Figure 5a,c,e). Fungal communities in annually burned plots were more stochastic (higher beta diversity) than those in biennially burned plots (*F*_1,38_ = 6.22, *p* = 0.02; Table 2 and Figure 4b), and had a greater abundance of *Mallochia* sp. (likely keratinophilic and coprophilous; Dale 1903; Ghosh et al. 1963; Ghosh 1985). Biennially burned fungal communities were less stochastic (lower beta diversity) and had higher abundances of *Staphylotrichum* sp. (dark brown pigmentation, thick spore walls; Ali et al. 2021; Table 4) and *Pleosporales* sp. (likely plant pathogen and/or saprotroph; Taylor et al. 2014). Fire regime treatments did not alter fungal alpha diversity; however (inverse Simpson; *F*_1,34_ = 0, *p* = 0.99; Table 5), both annual and biennial fire regime treatments had similar numbers of unique taxa (Figure 6). Furthermore, fungal communities were not correlated with burn patterns (Table 6). In summary, higher fire frequencies altered soil fungal community composition and resulted in greater beta diversity.

**TABLE 3:** PERMANOVA tables for fire frequency effects on fungal and bacterial communities.

Model term	D.F.	Sum Sqrs.	*R* ^2^	*F*	*p*	D.F.	Sum Sqrs.	*R* ^2^	*F*	*p*
		Fungi					Bacteria			
Fire regime	1	0.34	0.04	1.42	0.009**	1	0.07	0.03	1.26	0.09.
Residual	38	9	0.96			36	1.91	0.97		
Total	39	9.3	1			37	1.98			

*Note: p* < 0.1, *, *p* < 0.05; **, *p* < 0.01.

**TABLE 4:** Indicator species for annual and biennial fire regime treatments. The annual and biennial columns correspond to the relative abundance of the taxon in each fire regime treatment. Wi.ep is the Wilcoxon p‐value, which, for this analysis, only taxa with *p* < 0.001 were considered.

Kingdom	Fire regime	Taxonomy	Natural history notes	annual	biennial	wi.ep
Fungi	Annual	*Mallochia* sp.	Likely keratinophilic, often coprophilous	5.90	4.27	< 0.001***
	Biennial	*Staphylotrichum* sp.	Dark pigmentation, thick spore walls	7.66	9.24	< 0.001***
		*Pleosporales* sp.	Likely plant pathogen and/or saprotroph	3.90	6.31	< 0.001***
Bacteria	Annual	Gaiellales sp.	Aerobic	9.51	9.13	< 0.001***
		*Reyranella* sp.	Aerobic, gram negative	7.05	6.45	< 0.001***
	Biennial	*Streptomyces* sp.	Soil saprotrophs, found in coal fire sites	0.99	6.56	< 0.001***

*** *p* < 0.001.

**TABLE 5:** Linear mixed effect model tables for fire regime effects on soil microbial diversity (Inverse Simpson metric).

Model term	D.F.1	D.F.2	*F*	*p*	D.F.1	D.F.2	*F*	*p*
	Fungi				Bacteria			
Fire regime	1	34	0	0.99	1	32.34	0.02	0.88

Random effect	Variance	Std. Dev.	Variance	Std. Dev.
Field column	0	0	92.73	9.63
Residual	454.6	21.32	1836.23	42.85

*Note: p* < 0.1, *, *p* < 0.05; **, *p* < 0.01.

**TABLE 6:** Burn pattern component and diversity correlations with burn pattern and microbial community ordinations. Three PCoA axes are represented for the burn pattern and microbial ordinations to adequately describe variation in burn pattern (axes 1–3 describe > 75% of variation) and microbial community data (assessed with broken stick models to identify stopping points).

Model term		PCoA axes 1 & 2		PCoA axes 1 & 3		PCoA axes 2 & 3	
		*r* ^2^	*p*	*r* ^2^	*p*	*r* ^2^	*p*
Burn pattern	Unburned, green fuels	0.49	< 0.001***	0.16	0.04*	0.58	< 0.001***
	Black ash	0.32	< 0.001***	0.06	0.32	0.27	0.003**
	White ash	0.21	0.01**	0.26	0.004**	0.09	0.16
	Bare ground	0.07	0.26	0.31	< 0.001***	0.25	0.007**
	Grey ash	0.61	< 0.001***	0.51	< 0.001***	0.1	0.13
	Unburned, dead fuels	0.55	< 0.001***	0.29	< 0.001***	0.27	0.006**
Fungi	Unburned, green fuels	0.07	0.28	0.08	0.22	0.01	0.81
	Black ash	0.004	0.94	0.004	0.926	0.0004	1
	White ash	0.05	0.42	0.05	0.387	0.08	0.22
	Bare ground	0.03	0.62	0.02	0.739	0.03	0.62
	Grey ash	0.04	0.49	0.05	0.375	0.06	0.29
	Unburned, dead fuels	0.003	0.94	0.03	0.593	0.03	0.56
	Inverse Simpson diversity	0.36	0.001***	0.023	0.008**	0.14	0.07.
Bacteria	Unburned, green fuels	0.26	0.008**	0.28	0.006**	0.0387	0.49
	Black ash	0.08	0.23	0.08	0.26	0.0132	0.78
	White ash	0.1	0.16	0.09	0.2	0.1665	0.05*
	Bare ground	0.08	0.29	0.07	0.25	0.0764	0.25
	Grey ash	0.08	0.19	0.13	0.08.	0.0843	0.19
	Unburned, dead fuels	0.01	0.84	0.02	0.78	0.0119	0.81
	Inverse Simpson diversity	0.16	0.05*	0.02	0.64	0.18	0.04*

*Note: p* < 0.1, *, *p* < 0.05; **, *p* < 0.01; ***, *p* < 0.001.

### 3.1 Fire Regime Effects on Bacterial Communities

Fire regime treatments produced differences in soil bacterial communities, but this effect was not as strong as in fungal communities (*F*_1,36_ = 1.3, *p* = 0.09; Table 3 and Figure 5b,d,f). Bacterial communities in annually burned plots were more stochastic (*F*_1,36_ = 4.48, *p* = 0.04; Table 2 and Figure 4c), and had higher abundances of *Gaiellales* sp. (aerobic, gram negative; Table 4) and *Reyranella* sp. (aerobic, gram negative) relative to biennially burned treatments. Biennially burned bacterial communities, however, were less stochastic and had higher abundances of *Streptomyces* sp. (aerobic, gram positive, soil saprotroph, often associated with coal fires; Elshahawi et al. 2017). While the number of unique taxa was higher in annually burned plots (Figure 6), bacterial alpha diversity did not vary between fire regime treatments (*F*_1,32_ = 0.02, *p* = 0.88; Table 5). However, bacterial communities were correlated with burn patterns (Table 6). Specifically, the amount of unburned green fuel, grey ash and white ash were closely linked to bacterial community composition. In summary, post‐fire soil bacterial community composition was linked to variation in burn patterns and shifted with fire frequency, with higher frequency fires producing greater levels of beta diversity.

## 4 Discussion

Fire frequency driven increases in pyrodiversity altered soil microbial communities and increased microbial beta diversity. Annual fires resulted in greater pyrodiversity (more variation in burn pattern components between plots) than the biennially burned plots. The higher the degree of pyrodiversity in the annually burned plots was in part driven by patchier, the lower the severity fires (42.5% black ash—low severity, 13.4% grey ash—moderate severity, and 5.3% white ash—high severity), whereas higher severity fires in the biennially burned plots (20% black ash, 14.6% grey ash, and 28.1% white ash) contributed to more homogenous burn patterns. While fires were more severe in the biennial plots, burn pattern components like unburned fuels and bare ground did not vary between fire treatments (annual: 40%, biennial: 37%) implying that greater fuel build‐up in the biennially burned plots allowed for the more homogenous, higher severity burns. Despite greater fungal and bacterial beta diversity (stochasticity) in annually burned plots, only bacterial communities were directly linked to burn patterns. This may reflect shorter bacterial generation times that allow for rapid responses (less than 1 h) to fire severity effects on soils (Gibson et al. 2018; Weissman et al. 2021). While fungal community composition and diversity were not as closely linked to burn patterns, fungal beta diversity was still higher in annually burned plots, implying that indirect effects of fire on pyrodiversity (i.e., through soil abiotic conditions or plant communities) may mediate fungal pyrodiversity–biodiversity relationships (Gao et al. 2013; Mommer et al. 2018; Hewitt et al. 2022).

Soil bacterial and fungal responses to fire regime treatments demonstrate how different components of pyrodiversity influence belowground communities. Bacteria displayed rapid changes in community composition and beta diversity that were directly linked to heterogeneity in burn patterns. Since the annually burned plots had greater spatial variation in fire severity, this suggests that the rapid changes in bacterial communities and increases in beta diversity were the direct result of variation in fire conditions (Dao et al. 2022; Pulido‐Chavez et al. 2023). This is further supported by the greater abundance of the *Streptomyces* sp. in biennially burned plots. Members of *Streptomyces* are commonly found in soils near high severity, continuous coal fires, implying adaptations to surviving high temperatures (Elshahawi et al. 2017). Changes in fungal community composition and diversity were not linked to burn patterns; however, this implies the importance of other components of pyrodiversity, like changes in soil abiotic conditions or plant communities (Hart et al. 2005; Hopkins et al. 2021; Revillini et al. 2022). Since soil fungi, relative to bacteria, have slower growth rates (Rousk and Bååth 2011; Gostinčar et al. 2022) and are more closely associated with plant hosts (Fei et al. 2022; Kivlin et al. 2022), this may explain why fire frequency rather than burn heterogeneity was a stronger predictor of the changes in fungal communities. This is supported by the higher abundance of the *Staphylotrichum* sp. in biennially burned plots. Members of this genus produce dark, protective pigmentation (Lagashetti et al. 2019), which may help protect against the harmful effects of fire (Rajamani et al. 2021). Further, the *Mallochia* sp. that was more abundant in the annually burned plots is highly likely to be coprophilous (Dale 1903; Ghosh et al. 1963; Ghosh 1985). Coprophilous fungal taxa are known to proliferate after grassland fires (Wicklow 1975) and may be indicative of early, post‐fire taxa that opportunistically take advantage of post‐fire nutrient flushes (Peay et al. 2009; Pulido‐Chavez et al. 2023). Together, these indicator taxa demonstrate alternative pyrodiversity–related mechanisms that structure post‐fire fungal communities. Since soil microbes directly modify plant fuel loads (Hewitt et al. 2022; Gibb et al. 2022), it is likely that microbial pyrodiversity–biodiversity relationships also underlie the fire‐fuel interactions that structure fire regimes.

The close associations between plants and soil microbes mean that pyrodiversity–biodiversity interactions may underly the fire regimes of pyrophilic ecosystems. Spatial variation in fire characteristics produces ecosystem heterogeneity (i.e., pyrodiversity) through changes to soil abiotic characteristics (Steel et al. 2021; Jones and Tingley 2022). Since pyrodiversity also augments soil microbial beta diversity and ecosystem heterogeneity, this explains why plant diversity increases as well (He et al. 2019). This is supported by prior work demonstrating that pyrophilic plant species differ in germination and growth responses to fire severity related effects on soils (Revillini et al. 2022; Hopkins, Huffman et al. 2023; Hopkins and Bennett 2024b). Therefore, soil related mechanisms that boost plant diversity (in addition to natural successional changes) likely contribute to the high level of productivity required to generate the fuel loads that allow for recurrent fires (Platt et al. 2016; Archibald et al. 2018; Simpson et al. 2021). While fire frequency effects on plant community composition were not considered in this work, pyrodiversity does promote greater plant biodiversity (Ponisio et al. 2016; Miller and Safford 2020), implying that pyrodiversity effects on plants, microbes and soil characteristics can contribute to ecosystem productivity. This has important implications for land managers because finding the optimum frequency of prescribed fire that produces ecosystem heterogeneity should help achieve above and belowground diversity related restoration goals. More work testing the pyrodiversity–biodiversity hypothesis in other ecosystems with short fire return intervals like longleaf pine savannas (5 years), oak savannas (4 years), ponderosa pine forests (13 years) and short grass prairies (12 years) is required however, as climate change and anthropogenic influences are expected to alter fire frequency (Axelrod 1985; Hart and Hart 1997; Brockway et al. 2002; Jolly et al. 2015; Balch et al. 2017; Abrahamson 2023; Wasserman and Mueller 2023).

Identifying the optimum fire frequencies that promote pyrodiversity–biodiversity relationships will be key to predicting and responding to future effects of climate change and anthropogenic influence on ecosystems (Moritz et al. 2012; Keeley and Syphard 2016). In the grassland system used in this study, we found that annual fires promoted pyrodiversity–biodiversity relationships relative to biennial fire regime treatments. Tallgrass prairie ecosystems rely on combinations of fire (both natural and anthropogenic), grazing by bison and drier climates for their maintenance (Higgins 1984; Axelrod 1985; Robertson 2021). This suggests that anthropogenic suppression of fire that lengthens fire return intervals (Tveten 1997; DeSantis et al. 2011; Wagenius et al. 2020), rather than climate‐related increases in fire frequency, may have a greater impact on pyrodiversity–biodiversity relationships in grassland systems. In other pyrophilic ecosystems with naturally longer fire return intervals, however (e.g., ponderosa pine forests; 10–30 year intervals; Fitzgerald 2005), climate and anthropogenic influence on fire regimes should both be important. Specifically, shorter fire return intervals caused by longer fire seasons and/or anthropogenic ignition could result in reduced fuel loads that produce less variable, low severity fire (Agee 1993; Kay 2000; Banerjee et al. 2020). Alternatively, longer fire return intervals in pyrophilic forests caused by fire suppression can lead to larger fuel loads that produce spatially homogenous, stand‐replacing fires (Kolden 2019; Roos et al. 2020). Therefore, optimum fire frequencies in pyrophilic forests should be those that allow for spatial variation in fire severity, while not driving mass mortality of under‐and overstory communities.

In summary, changes in fire frequency alter microbial communities and beta diversity through pyrodiversity‐related mechanisms. This work is the first to link fire frequency effects on pyrodiversity to changes in soil microbial biodiversity and shows that microbial responses to pyrodiversity vary between bacteria and fungi. By manipulating fire frequency, we demonstrated that higher frequency fire produced more heterogeneous burn patterns and greater microbial beta diversity in a grassland ecosystem. Since bacterial and fungal responses to pyrodiversity differ, future work should test how pyrodiversity effects on soil microbial communities are linked to changes in soil abiotic conditions, plant communities, and ecosystem productivity. Additionally, more work is required to identify the optimum fire regime conditions that promote pyrodiversity–biodiversity relationships in other ecosystems. To conclude, fire produces ecosystem heterogeneity and modifies soil microbial communities and biodiversity, but this effect is modified by fire frequency.

## Author Contributions

Jacob R. Hopkins developed and designed the experiment. Jacob R. Hopkins and Thomas P. McKenna established the field site and managed the prescribed burns. Jacob R. Hopkins and Thomas P. McKenna performed the field portion of the research. Jacob R. Hopkins performed the lab work and analysed the data. All authors wrote the manuscript.

## Disclosure

Benefit‐Sharing: A research collaboration was developed with scientists from the Land Institute; all collaborators are co‐authors. The results of the research have been shared with the Land Institute and the broader scientific community (see above). This research addresses priority concerns, in this case, the application of prescribed fire management to tallgrass prairie and grassland ecosystems. Further, our group is committed to international scientific partnerships and providing the results of this research to all interested parties through personal communication and via publicly accessible databases (described above).

## Conflicts of Interest

The authors declare no conflicts of interest.

## Supporting information


Data S1.

## Data Availability

Raw sequence reads and metadata are deposited in the SRA (PRJNA1240331 and PRJNA1241203). Metadata and all other data relevant to this work are available on Dryad: http://datadryad.org/stash/share/E5k7ATNOfncw9_TyCmo3bgBPVH4HkT4A6K‐86C1Wkko, DOI: 10.5061/dryad.kprr4xhf4.
