# Supplementary material for: Fire Frequency Driven Increases in Burn Heterogeneity Promote Microbial Beta Diversity: A Test of the Pyrodiversity‐Biodiversity Hypothesis
Source: Mol Ecol. 2025 Apr 5;34(10):e17756. doi: 10.1111/mec.17756 (PMC12051778; doi:10.1111/mec.17756)
Supplement: Supplementary file 1 — Data S1. [file MEC-34-e17756-s001.pdf]

MOLECULAR ECOLOGY

Supplemental Information for:

Fire frequency driven increases in burn heterogeneity promote microbial beta diversity: a test of the pyrodiversity-biodiversity hypothesis

Jacob R. Hopkins, Alison E. Bennett, and Thomas P. McKenna

Table of Contents:

**Site management information**	Page 2-3
**Bioinformatics**	Pages 4-6

MOLECULAR ECOLOGY

Site management information

Table S1: Species list for original seed mix. Present refers to presence in site as of Fall 2024.

Genus	Species	Common name	Functional group	Present
Callirhoe	*digitata*	Fringed poppy mallow	forb	no
Coreopsis	*tinctoria*	Plains coreopsis	forb	yes
Coreopsis	*tripteris*	Grand coreopsis	forb	yes
Echinacea	*pallida*	Pale purple coneflower	forb	yes
Eryngium	*yuccifolium*	Rattlesnake master	forb	yes
Heliopsis	*helianthoides*	Ox-eye sunflower	forb	yes
Liatris	*pycnostachya*	Prairie blazing star	forb	no
Monarda	*fistulosa*	Wild bergamot	forb	yes
Penstemon	*digitalis*	White beardtongue	forb	no
Physostegia	*virginiana*	Fall obedient plant	forb	yes
Pycnanthemum	*tenuifolium*	Slender mountain mint	forb	no
Pycnanthemum	*verticillatum var. pilosum*	Hairy mountain mint	forb	yes
Ratibida	*pinnata*	Gray-headed coneflower	forb	yes
Rudbeckia	*hirta*	black-eyed susan	forb	yes
Senna	*hebecarpa*	Wild senna	forb	yes
Silphium	*integrifolium*	Rosin weed	forb	yes
Solidago	*nemoralis*	Gray goldenrod	forb	yes
Solidago	*speciosa*	Showy goldenrod	forb	yes
Symphyotrichum	*pilosum*	Frost aster	forb	yes
Verbena	*stricta*	Hoary vervain	forb	yes
Verbesina	*alternifolia*	Yellow wingstem	forb	yes
Vernonia	*baldwinii*	Western ironweed	forb	no
Agrostis	*hyemalis*	Winter bentgrass	gramminoid	yes
Andropogon	*ternarius*	Split beard	gramminoid	yes
Andropogon	*virginicus*	Broomsedge	gramminoid	yes
Carex	*praticola*	Meadow sedge	gramminoid	yes
Panicum	*capillare*	Witchgrass	gramminoid	yes
Schizachyrium	*scoparium*	Little blue	gramminoid	yes
Sphenopholis	*obtusata*	Prairie wedge grass	gramminoid	yes
Amorpha	*canescens*	Lead plant	legume	yes
Baptisia	*alba*	White indigo	legume	yes
Chamaecrista	*fasciculata*	Partridge pea	legume	yes

MOLECULAR ECOLOGY

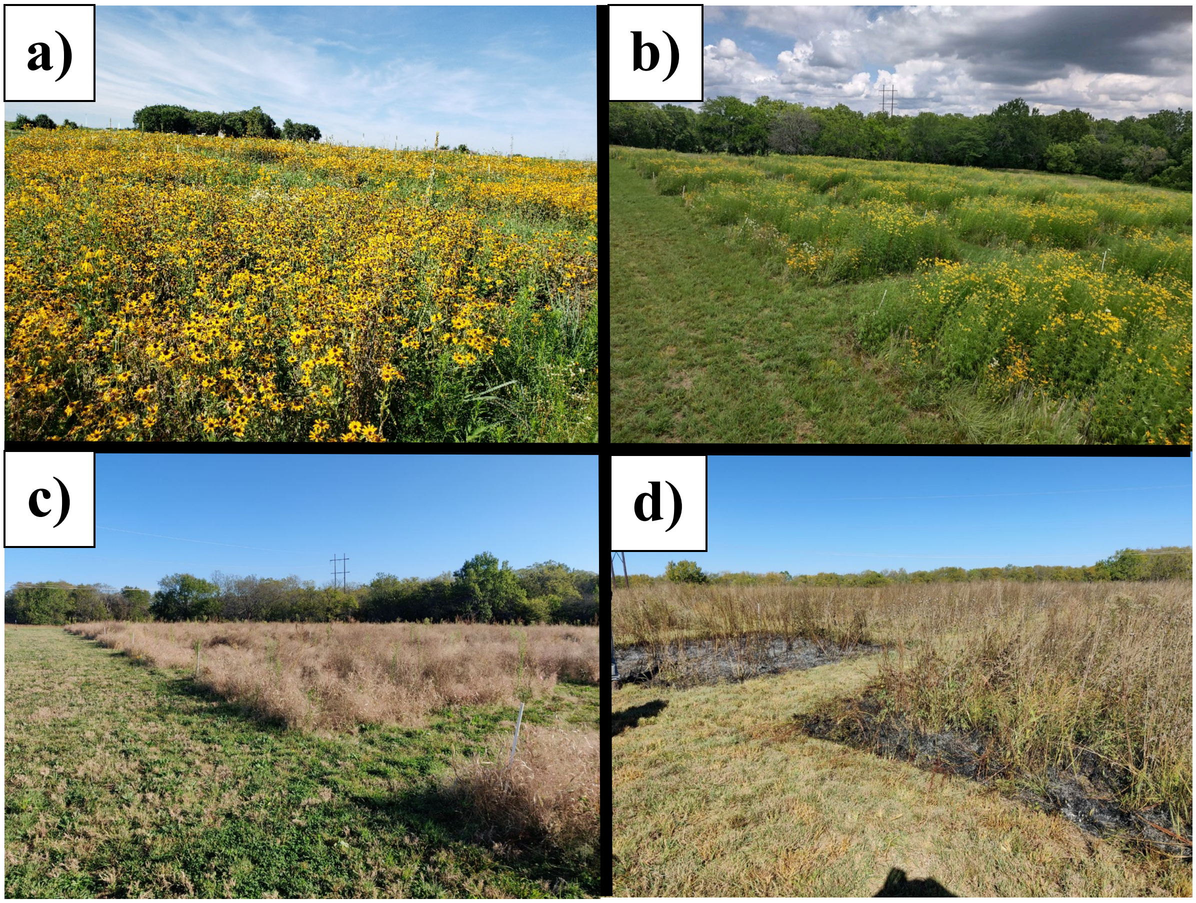

Figure S1: Site pictures of plots and plant communities. Plant community composition has changed across time with overall plot biomass increasing and shifts in dominance from a) *R. hirta*, *C. tinctoria*, and *R. pinnata* in summer 2022, to b) greater cover by graminoids and other forms like *M. fistulosa*, *P. verticillatum var. pilosum*, and *S. pilosum* in summer 2023 and 2024. C) Fuel loads do vary between the fire regime treatments, with greater amounts of dead fuels in biennially burned relative to annually burned plots. D) the variation in plant fuel loads produce distinct burn patterns with biennially plots producing higher severity, homogenous burns (left plot in picture) and annual fires producing lower severity, more heterogeneous burn patterns (right side of the picture). Despite greater heterogeneity in annual burn patterns, fire lines carry through the entire plot and remove 60-80% of dead fuels.

MOLECULAR ECOLOGY

Bioinformatics

1. Inspect your metadata
    a. qiime tools inspect-metadata [filename]
    b. qiime metadata tabulate --m-input-file sample-metadata.tsv --o-visualization tabulated-metadata.qzv
        i. creates a visualization that can be viewed in QIIME2 viewer: https://view.qiime2.org/

2. Import FASTQ files as QIIME2 artifact
    a. qiime tools import --type SampleData[PairedEndSequencesWithQuality] --input-path [directory w/ fastq files] --output-path reads.qza --input-format CasavaOneEightSingleLanePerSampleDirFmt

3. Visualize the sequence results
    a. qiime tools peek reads.qza
    b. qiime demux summarize --i-data reads.qza --o-visualization demux.qzv

4. now come the trimming steps:
    a. qiime cutadapt trim-paired \
    --i-demultiplexed-sequences reads.qza \
    --p-front-f [forward primer code here]\
    --p-front-r [reverse primer code here]\
    --o-trimmed-sequences demux-trimmed.qza

    b. qiime dada2 denoise-paired \
    --i-demultiplexed-seqs demux-trimmed.qza \
    --p-trim-left-f 0 \
    --ptrim-left-r 0 \
    --p-trunc-len-f [258 bacteria, 269 fungi] \
    --p-trunc-len-r [216 bacteria, 219 fungi] \
    --o-representative-sequences rep-seqs-dada2.qza \
    --o-table dada2_table.qza \
    --o-denoising-stats dada2_stats.qza

        i. qiime feature-table summarize \
        --i-table dada2_table.qza \
        --o-visualization dada2_table.qzv \
        --m-sample-metadata-file [metadata_file.tsv]

ii. qiime feature-table tabulate-seqs \
--i-data rep-seqs-dada2.qza \
--o-visualization rep-seqs-dada2.qzv

iii. qiime tools export \
--input-path dada2_output/denoising_stats.qza \
--output-path dada2_output_output

5. export the OTU table
   a. qiime tools export --input-path dada2_table.qza --output-path exported_otu_table
   b. biom convert -i exported_otu_table.biom -o fungi_otu_table.txt --to-tsv

6. filter OTU table
   a. remove OTUs that occur less than 5 times
      i. qiime feature-table filter-features \
      --i-table dada2_table.qza \
      --p-min-frequency 5 \
      --o-filtered-table feature_filtered_table.qza
   b. remove samples with < X reads
      i. qiime feature-table filter-samples --i-table feature_filtered_table.qza --p-min-frequency X --o-filtered-table sample_freq_filtered_table.qza

7. prepare taxonomic data
   a. go to: https://unite.ut.ee/repository.php
      i. click on the QIIME release tab
      ii. as of now, select click the link for 8.0 "includes singletons set as RefS (in dynamic files)
      iii. download these, then add them to your cluster folder
   b. qiime tools import --type FeatureData[Sequence] \
   --input-path sh_refs_qiime_ver8_dynamic_02.02.20.fasta \
   --output-path unite_ver8_dynamic_seqs.qza
   c. qiime tools import --type FeatureData[Taxonomy] \
   --input-path sh_taxonomy_qiime_ver8_dynamic_02.02.2019.txt \
   --output-path unite_taxonomy_ver8.qza \
   --input-format HeaderlessTSVTaxonomyFormat

8. train the classifier, note that SILVA classifier has already been prepared
   a. qiime feature-classifier fit-classifier-naïve-bayes \
   --i-reference-reads unite_ver8_dynamic_seqs.qza \
   --i-reference-taxonomy unite_taxonomy_ver8.qza \
   --o-classifier unite_ver8_dynamic_classifier.qza

9. assign taxonomy
    a. qiime feature-classifier classify-sklearn \
       --i-classifier unite_ver8_dynamic_classifier.qza \
       --i-reads dada2-rep-seqs.qza \
       --o-classification taxonomy.qza
    b. qiime taxa barplot \
       --i-table dada2_table.qza \
       --i-taxonomy taxonomy.qza \
       --m-metadata-file mapping/metadata.tsv \
       --o-visualization taxa_barplots.qzv

10. combine the taxonomy file with filtered OTU table
    a. qiime tools export \
       --input-path taxonomy.qza \
       --output-path exported_taxonomy.table
